# Dental Applications of Ion-Substituted Hydroxyapatite: A Review of the Literature

**DOI:** 10.3390/dj12100304

**Published:** 2024-09-25

**Authors:** Eisha Imran, May L. Mei, Kai Chun Li, Jithendra Ratnayake, Manikandan Ekambaram, Paul R. Cooper

**Affiliations:** Sir John Walsh Research Institute, Faculty of Dentistry, University of Otago, Dunedin 9016, New Zealand; may.mei@otago.ac.nz (M.L.M.); kc.li@otago.ac.nz (K.C.L.); jithendra.ratnayake@otago.ac.nz (J.R.); mani.ekambaram@otago.ac.nz (M.E.)

**Keywords:** hydroxyapatite, calcium phosphates, remineralization, antibacterial, antibiofilm, ionic substitution, caries management, hardness, biocompatibility, dental applications

## Abstract

Hydroxyapatite (HA) forms an essential constituent of human teeth and bone. Its distinctive characteristic features, such as bioactivity and osteoconductivity, make it an ideal candidate to be used as an implant coating in restorative dentistry and maxillofacial surgery for bone regeneration. However, low fracture toughness and brittleness are a few of the inherent features of HA, which limit its application in load-bearing areas. The potential of HA to engage its lattice structure with either partial or complete substitution with external ions has become an increasing area of research as this phenomenon has the potential to enhance the biological and functional properties of the material. Consequently, this review aimed to highlight the role of various substituted ions in dental applications. Data indicate that the newly formed HA-substituted biomaterials demonstrate enhanced remineralization and antimicrobial activity along with improved hardness. Ion-substituted HA offers a promising strategy for future clinical research as these materials may be incorporated into various dental products for therapeutic treatments.

## 1. Introduction

**Figure 1:**
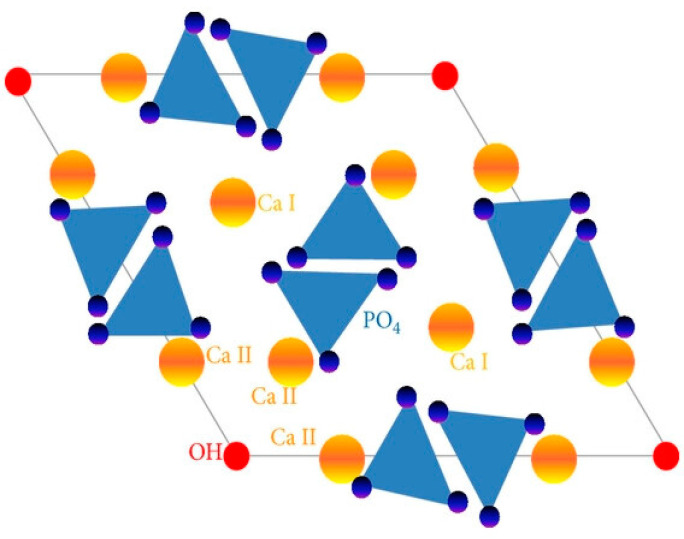
Basic structure of hydroxyapatite crystal unit showing the structural locations of the constituents [6].

The core ingredient of enamel and dentin consists of platelet-like crystals of hydroxyapatite (HA), which forms a crucial building block of the mineral phase and is mainly constituted of calcium and phosphate [1]. Indeed, enamel rods consist of tightly packed hydroxyapatite (HA) crystals that provide strength and rigidity to the tissue [2], while in dentin, the HA crystals are present in the form of flattened plate structures. Pure stoichiometric HA has a Ca/P mole ratio of ~1:67 (39.68 wt% calcium and 18 wt% phosphorus) and is characterized as highly stable and insoluble [1]. A typical lattice structure of HA is represented as (Ca_10_(PO_4_)_6_OH_2_). It has a unique structure that consists of an OH^−^ group in a columnar structure, which is present at the edge of the elementary cell (Figure 1). The distribution of oxygen atoms in the group hinders the formation of hydrogen bonds [3]. Ca (I) and Ca (II) are the two types of calcium cations present in the HA lattice structure. Ca (I) is enveloped around six oxygen atoms that originate from four orthophosphate groups, while Ca (II) is encompassed around seven oxygen atoms, emerging from four orthophosphate groups along with one hydroxyl ion [4,5]. The Ca-O distance in Ca (I) is longer (bond length of 0.255 nm) in contrast to Ca (II) (bond length of 0.245 nm), as documented by crystallographic investigations. Fortified by the difference in bond length, the Ca (II) sites are energetically favorable for smaller-sized cations in contrast to Ca (I) sites, which accommodate larger cations [4]. Figure 1 demonstrates the basic structure of a hydroxyapatite crystal unit. The atoms of Ca (I) are located at the outer edges of the HA crystal unit, while Ca (II) atoms are present in the center along with the OH^−^ group, forming an equilateral triangle [6]. The largest ions present within the lattice structure are the phosphate ions and they predominantly determine the structure of the lattice [5].

HA is characterized as a non-toxic, bioactive, and osteoconductive material that has the potential to directly form chemical bonds when in contact with living tissues [7]. These features make it an ideal candidate for use as an implantable material in maxillofacial surgery, dentistry, and orthopedic surgery for bone repair defects or coating materials for use as implants [1]. The thermal stability of HA is also an essential parameter as this greatly affects its morphology, mechanical properties, solubility, and biocompatibility [1]. In recent decades, synthetic HA has been widely utilized for biomaterial applications, and this necessitates a thorough understanding of its properties before it can be synthesized and incorporated into commercial products [8]. Consequently, the development of HA products with enhanced biological and therapeutic properties has attracted significant interest from both researchers and clinicians [9,10]. Numerous studies have now focused on using biogenic sources to produce HA as these provide several advantages such as relatively low cost, easy accessibility, and generation of fewer byproducts [9,11]. Moreover, studies have shown that HA produced from biogenic sources is able to preserve the natural framework of bone. Animal bones, eggshells, and fish scales are examples of several the biogenic sources reported in the literature [8,12]. However, low fracture toughness and brittleness are a few of the immanent features of HA that limit its application under load bearing when placed within the human body [7]. Notably, HA is also slowly resorbed in vivo when implanted in the body [13]. Interestingly, a key characteristic of HA is its ability for ionic exchange/substitution. Inorganic tooth components include essential trace elements, such as magnesium (Mg^2+^), iron (Fe^2+^), zinc (Zn^2+^), sodium (Na^+^), silicate (SiO_4_^4−^), and carbonate (CO_3_^2−^). Therefore, to produce a mineral component similar to natural bone or teeth with enhanced properties, laboratory studies have aimed to incorporate various ion substitutions into synthetic HA. This process of ion substitution has been demonstrated to alter the crystal structure of the HA lattice, which, in turn, affects the material morphology, solubility, and thermal stability [14]. Differences in ionic radius or charge determine the type of exchange, which can either be complete or partial. Complete exchange is theoretically possible when the exchange of ions is with the same charges, such as OH^−^ substituted with (fluoride) F^−^ or Ca^2+^ substituted with (strontium) Sr^2+^ [6]. However, the exchange of ions with different charges, such as PO_4_^3−^ with CO_3_^2−^, creates a vacancy that simultaneously releases one Ca^2+^ and one OH^−^ [15]. Various cations, such as Sr^2+^, Mg^2+^, or (lead) Pb^2+^, exhibit the same oxidation state as Ca^2+^ in the crystal lattice structure [16], while anions, such as F^−^ or Cl^−^, occupy the same state as OH^−^ [6]. In the last decade, a significant number of studies have been published regarding the role of HA or nano-HA, allowing for a better understanding of its properties and applicability in medicine and dentistry. In the last decade, a significant number of studies have been published regarding the role of HA or nano-HA, allowing for a better understanding of its properties and applicability in medicine and dentistry [17,18,19]. Indeed, various reviews have evaluated the efficacy of substituted HA or nanoparticles in bone regeneration [14,20] or biomedical applications [21]. However, to date, no review has been specifically undertaken to evaluate the broad scope of substituted HA in dental applications. Therefore, this review aims to be the first to provide an overview of the current literature on the applicability and efficacy of various substituted ions in HA for dental applications.

## 2. Literature Search Strategy

A literature search was conducted using PubMed from January 2010 to December 2023 using the following MesH keywords: hydroxyapatite strontium OR hydroxyapatite zinc OR hydroxyapatite magnesium OR hydroxyapatite silver OR hydroxyapatite fluoride OR (hydroxyapatite silicone OR hydroxyapatite copper OR hydroxyapatite cobalt OR hydroxyapatite manganese OR hydroxyapatite Iron OR hydroxyapatite cerium) AND (dentistry OR dental applications). The titles and abstracts were screened, and manuscripts were included using the following criteria described below.

### 2.1. Inclusion Criteria

Laboratory studies;

Randomized and non-randomized clinical trials.

Studies on anionic or cationic substitution with hydroxyapatite;

Studies related to dental applications;

Open-access articles.

### 2.2. Exclusion Criteria

Studies on hydroxyapatite without any ionic substitution;

Studies not applicable in dentistry;

Studies in a language other than English;

Review articles;

Case reports;

Conference papers, letters, editorials, opinions, and book chapters.

**Figure 2:**
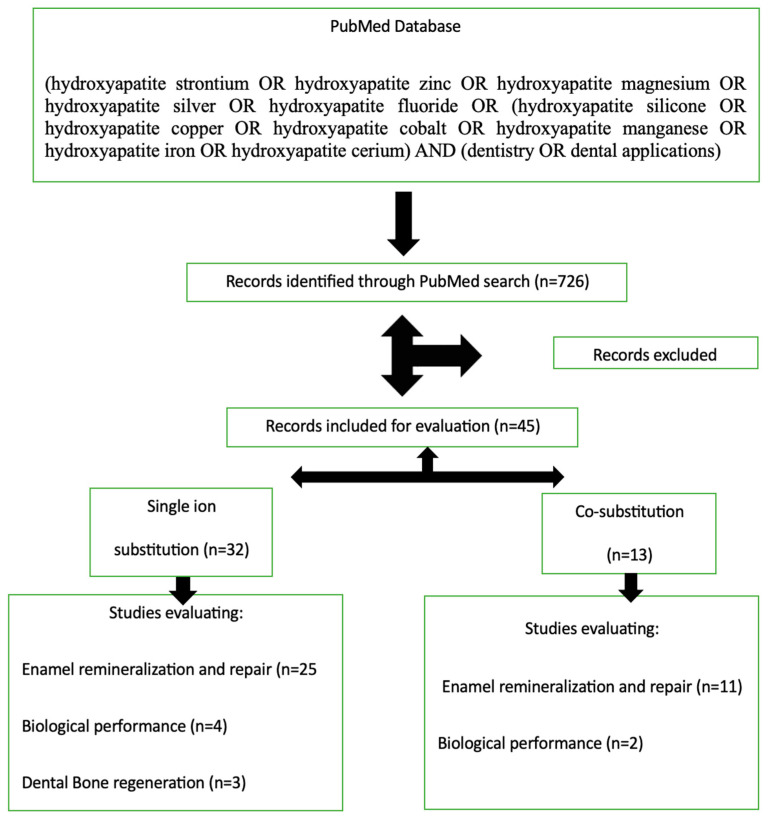
Flowchart of the study selection used for identifying the literature used in this review.

Consequently, seven hundred and twenty-six articles were identified initially. After abstract evaluation, a total of forty-five were included in this study. Figure 2 below depicts a schematic representation of the selection process.

## 3. Ionic Substitution in the HA Crystal Lattice Structure

**Figure 3:**
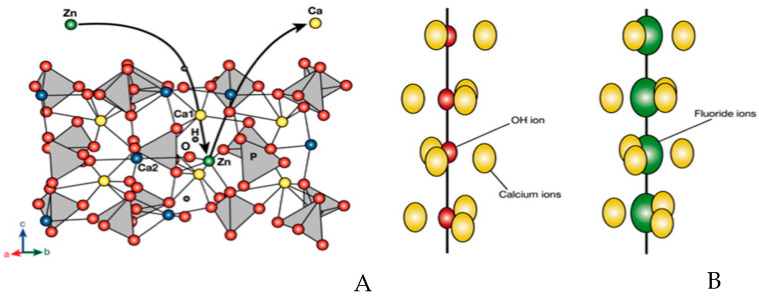
Substitution types in the HA lattice. (**A**) Cationic substitution. (**B**) Anionic substitution [14].

Ionic substitution can occur in the HA crystal structure and can significantly enhance its properties by lowering the ion release rate and prolonging its effectiveness [6]. This process is driven by ionic charge, leading to either cationic or anion substitution. Notably, the calcium sites in the HA lattice can be replaced with Mg^2+^, Ag^+^, or Zn^2+^, a process known as cationic substitution. Ionic substitution involves replacing OH^−^ with either F^−^ or Cl^−^ or substituting the phosphate group with carbonate or silicate groups (Figure 3) [14].

The potential of HA to engage its lattice structure with either partial or complete substitution with external ions allows for the imparting and augmentation of new properties in the material. Indeed, an essential parameter that needs to be considered is maintaining the crystallographic integrity of the lattice as the stoichiometric ratio of Ca/P may decrease from 1.6 to 1.33 after successful substitution on the sites occupied by the Ca^2+^, or it might increase to 1.85 if substituted with phosphate sites [22]. The creation of non-stoichiometric defects, in vacant sites, aids in accommodating the external ions or atomic species within the lattice structure and, as a result, produces extrinsic, as well as intrinsic, effects [23]. A pure HA analog displays poorer crystallinity in contrast to its substituted structure, and this can be explained by the fact that the accumulation of defects creates distortions within the lattice structure. Notably, this reduces its crystallinity and simultaneously increases its solubility [24]. The newly generated substituted structure, if placed in a biological environment, demonstrates modification of various properties, including porosity, mechanical response, geometry, or even cell adhesion [24]. Along with these intrinsic effects, several external effects may also arise such as hydration energy and modification of nucleus charge density [24]. These effects are likely to modify the crystal growth or nucleation, therefore altering the material properties. However, only a limited number of studies on this have been reported in the literature due to the dynamic nature of the process, while current characterization techniques have been mainly focused on analyzing static structures [24].

### 3.1. Ionic Substitutions in HA for Dental Applications

In dentistry, ionic substitutions of HA have more recently gained widespread interest as they endeavor to generate materials with enhanced properties, such as increased mechanical, antimicrobial, or osteo-induction activity. Notably, the introduction of substitutions in sub-lattices (anionic or cationic) augments the bioactive behavior of (HA) particles, as depicted in Figure 3. Cationic substitution occurs when Ca^2+^ in the HA structure is partially replaced with ions such as Zn^2+^, Mg^2+^, or Ag^+^, resulting in a decrease in the “a” axis along with an increase in the “c” axis (Figure 3A). Anionic substitution is when a smaller ion (OH^−^) is replaced with a larger ion (F^−^) (Figure 3B) [14].

The crystalline nature of the HA structure allows for the ion incorporation process to be relatively straightforward and utilizes mechanisms that are substitutive or interstitial [25] with a broad overview of dental applications, as highlighted in Table 1 and Table 2. This indicates that Zn^2+^ was used most frequently for substitution followed by Mg^2+^, Ag^+^, and F^−^. Other ions that have been used for substitution include Sr^2+^, Si^4+^, Ce^3+^, Fe^2+^, and CO_3_^2−^.

Most of the commonly used substituted ions in the studies identified were cations. This is because calcium has a greater tendency to be substituted in contrast to phosphate as these ions are relatively smaller in size, more mobile, and more easily fill the voids in the crystal lattice [24]. In addition, the retention and release of calcium is more favorable in contrast to phosphates, which may form tight hydration spheres as the protons of the water molecules are strongly attracted toward the negatively charged phosphate group [24,26].

#### 3.1.1. Zinc

The importance of Zn^2+^ has been published in the literature as an essential trace element; therefore, it has attracted the attention of many researchers over the past few decades [25]. Many enzymes containing zinc are responsible for metabolic conversion as hydrolases for the degradation of various proteins, lipids, and nucleic acid. Moreover, zinc plays an important role in the structural function, growth, and development of tissues, and is also involved in various processes, such as cell division or gene transcription [27]. Consequently, its deficiency can result in a compromised immune system and serious pathologies. The isoelectric Zn^2+^ substitution of Ca^2+^ in the lattice structure results in a decrease in lattice parameters due to changes in ionic radius. However, only limited quantities of zinc (20 atom%) can be incorporated into the HA lattice structure. Numerous studies have documented the antibacterial properties of zinc against Gram-positive and Gram-negative bacteria when substituted within HA, and they have been also found to have other efficacious bioactivity [20,28]. As detailed in Table 1 and from a review that summarized the action of zinc hydroxyapatite on remineralization, it can be concluded that zinc can be widely used as a remineralization agent. In addition, Zn^2+^ substituted HA reduces bacterial adherence and the dental calculus deposition [29,30].

#### 3.1.2. Silver

Ag^+^ is not normally found in the human body unless there is supplementation or contamination. Notably, the antibacterial activity of Ag^+^ has gained widespread attention from many researchers, and it is now frequently included in HA coatings of biomaterials to prevent infections at the dental implant site and during orthopedic surgeries [20]. Ag^+^ binds to microbial DNA, therefore preventing replication. Interestingly, several studies have also documented the limited capacity of microbes to develop resistance to Ag^+^ in contrast to all other metal ions. Indeed, Ag^+^ has demonstrated the highest antibacterial potential even at relatively low concentrations, such as at 35 ppb. Notably, Ag^+^ can often result in a black coloration of tissues when oxidized [31], compromising aesthetics when applied in dental procedures.

#### 3.1.3. Magnesium

Magnesium is important in skeletal formation and cell membrane stabilization. Indeed, 30 g of magnesium is normally found in an adult weighing 70 kg, and its deficiency may result in compromised physical and mental growth [32]. As highlighted in Table 1, Mg^2+^ substitution in HA was reported in two of the studies identified in this study. In one study, Mg^2+^ utilized in combination with Zn and F from a commercially obtained toothpaste demonstrated enhanced enamel remineralization, and the formation of a new crystalline phase in the enamel was observed along with dentinal tubular occlusion. The compositional analysis indicated that Mg^2+^ was used in combination with other ions, so the sole effect of Mg^2+^ could not be accurately ascertained [33]. However, in an additional study, the remineralization and antibacterial properties of Mg-HA dental adhesives were investigated, and a significant increase in bond strength, the formation of a thicker hybrid layer, and a decrease in the percentage of live bacteria was observed as the concentration of incorporation of Mg^2+^ increased [34].

#### 3.1.4. Iron

The main role of Fe^2+^ is in oxygen transportation as it is an essential component of hemoglobin. Furthermore, Fe^2+^ is required along with other reducing agents during the process of collagen synthesis. The substitution of Fe^2+^ affects the biological and physiochemical properties of HA without demonstrating any signs of cytotoxicity. Fe^2+^ substituted HA also demonstrates improved bioactivity, which subsequently results in the formation of an apatite layer on titanium surfaces and ultimately enables improved bonding. Furthermore, an in vitro study by Rau et al., which utilized Fe^2+^ substitution into the HA crystal lattice structure, augmented the rate of proliferation of osteoblasts at the site of implant placement [35].

#### 3.1.5. Silicate

Due to its significant bioactivity in physiological processes, silicate (Si^4+^) is widely used as a substitutional element. It is found in a similar concentration to that of Zn, Cu, and Fe^2+^ in the human body, and it is an essential trace element playing a significant role in skeletal development and repair [36]. Moreover, it is also known to play metabolic roles along with a structural role as a stabilizer of collagen networks and as a biological cross-linker [37,38,39]. Khonina et al. evaluated the remineralization effects of Si-HA-glycerohydrogels using two different HA concentrations, and the data indicate that there was increased hardness in the extracted tooth samples treated with the substituted materials [40].

#### 3.1.6. Strontium

Dental applications involving strontium (Sr^2+^) substituted HA are mainly used to enhance the material’s bioactivity and physicochemical properties [41]. Better solubility was observed when Sr^2+^ was partially substituted into HA at the Ca lattice sites in contrast to pure HA [42]. In vitro studies have documented enhanced mechanical and antibacterial properties when HA is used in combination with Sr^2+^. Moreover, biocompatibility analysis has revealed no cytotoxic effects of Sr^2+^ [43,44].

#### 3.1.7. Cerium

In a study by Shahid et al., the effects of Cerium (Ce^3+^) were analyzed, and it was concluded that Ce^3+^ substitution concentrations had a directly proportional effect on antibacterial activity. Moreover, the newly formed substituted biomaterial also demonstrated excellent mechanical properties when assessed by Vickers hardness testing [45].

#### 3.1.8. Carbonate

Along with Ca^2+^ and PO_4_^3−^, CO_3_^2−^ are the most abundant ions, constituting HA, and they are 2–8% of the inorganic component in the bone structure consequently giving the same composition as a natural bone framework. CO_3_^2−^ can be used as a substitute material in tissue engineering because of its higher resorption rates and improved mechanical properties in contrast to pure HA particles. A study using CO_3_^2−^ in combination with Ag^+^ demonstrated that the newly synthesized material exhibited beneficial effects in preventing implantitis [46].

#### 3.1.9. Fluoride

Fluoride plays an important role in developing and sustaining oral hygiene [47]. In the oral cavity, the availability of fluoride in ionic form has the ability to promote remineralization by a variety of mechanisms, including enhancing the crystallization kinetics, therefore increasing the mineralization process while solubility of the apatite phase is also reduced in the process [48]. Moreover, fluoride also inhibits the enzyme activity in oral bacteria, thereby providing antibacterial action that can also limit carious disease progression. Fluorohydroxyapatite (FHA) is formed by the substitution of the OH^−^ present in the mineral component of teeth with the F^−^ [49]. Moreover, FHA can also be synthesized using synthetic HA or HA isolated from natural sources [50]. The overall aim of substitution is to provide the new scaffold with superior mechanical properties, which is achieved by increasing the crystallinity and size of HA crystal along with a reduction in solubility [51]. Studies have also signified that FHA exhibits superior biological properties in contrast to HA [49,52]. A fewer number of dead cells along with better cell proliferation can be observed [52,53]; however, it is pertinent to control the concentration of F- in the lattice structure conducive to attain optimum bioactivity results.

**Table 1:** Details of the studies and applications of HA-substituted ions in dentistry.

Ion Substituent	Aim of the Study	Dental Applications	Outcomes	Reference
**Iron (Fe^2+^)**	To provide enhanced properties such as osteointegration for obtaining a tight fixation between the bone and dental implant. Fe^2+^ was deposited at the HA coating on Ti dental implants.	Biomaterial to enhance osteointegration between the dental implant and bone.	**Mechanical properties:** Vickers hardness testing was used to evaluate the hardness at the film/substrate interface. Improved surface hardness values were obtained after exposing the sample to the newly formed substituted structure.	[35]
**Zinc (Zn^2+^)**	To evaluate the efficacy of Zn-HA paste on sites affected by molar–incisor hypomineralization (MIH) and control teeth (no treatment).	Toothpaste for enamel remineralization.	**Remineralization:** MIH treatment needs index and the Schiff Air index for sensitivity: Zn-HA paste demonstrated statistically improved scores after 9 months of treatment.	[54]
**Zinc (Zn^2+^)**	To evaluate the protective efficacy of Zn-HA toothpaste in response to the erosion caused by soft drinks and to compare it with F toothpaste.	Toothpaste to prevent erosion.	**Remineralization:** SEM analysis demonstrated the Zn-HA paste provided better enamel protection when compared with the F group.	[55]
**Zinc (Zn^2+^)**	To develop and synthesize a Zn-HA biomaterial for the prevention of enamel demineralization during the cold-light bleaching procedure. The following groups were used for comparison: HA. Zn-HA (2%, 4%, and 8% Zn). Deionized water.	Biomaterial for enamel remineralization.	**Remineralization:** XRD results demonstrated an increase in the crystallinity of the HA and Zn-HA group after treatment. SEM analysis revealed a smoother surface after treatment in both the test groups. **Biological performance:** Cell response: CCK-8 cytotoxicity analysis was used to evaluate the cytotoxicity of the different concentrations of Zn-HA. The 1% Zn-HA demonstrated no toxic response to cells in vitro; however, higher concentrations revealed cell shrinkage and death. Antibacterial response: The concentration of Zn was directly linked to its antibacterial activity.	[56]
**Zinc (Zn^2+^)**	To compare and evaluate the difference in the remineralization of various available toothpastes: Fluoridated. Herbal. Zn-HA. Calcium sucrose phosphate.	Toothpaste for remineralization.	**Remineralization:** SEM analysis was used to evaluate the remineralising effect on the depth of demineralized enamel lesions. Zn-HA toothpaste demonstrated the highest remineralization effect in contrast to the other groups.	[57]
**Strontium (Sr^2+^)**	To develop and evaluate the remineralization potential of a Sr-HA paste for caries management.	Toothpaste for remineralization.	**Remineralization:** SEM analysis was used to analyze the surface topography. EDX analysis was performed to compare the Ca and P levels with a comparison undertaken with sound enamel. The mean values of Ca and P were significantly higher in the group treated with the Sr-HA paste in contrast to the sound tooth.	[58]
**Magnesium (Mg^2+^)**	To use Mg-HA for alveolar ridge preservation as a grafting material and to determine the modification in protein expression along with the gene activation involved in bone metabolism.	Grafting material to preserve the alveolar ridge.	**Bone metabolism markers:** Markers for bone catabolism were activated. An increase in bone remodeling tissue was obtained in contrast to the baseline.	[59]
**Strontium (Sr^2+^)**	To develop and evaluate the remineralization potential of Sr-HA and to compare it with casein phospho-peptide (CPP-ACP)	Dentifrice for remineralization.	**Remineralization:** Remineralization was evaluated on the basis of SEM and EDX analysis. It was concluded that Sr-HA demonstrated significantly higher remineralization in contrast to CPP-ACP toothpaste.	[60]
**Magnesium (Mg^2+^)**	To evaluate the regeneration of the alveolar ridge (socket preservation) after treatment with Mg-HA.	As a grafting material for the preservation of sockets.	**Remodeling of a collagen matrix.** Histological analyses were performed and observed under a polarized and light microscope. New bone formation was observed without any signs of inflammation. Remodeling of collagen initiated from apical region toward the coronal end and complete regeneration was observed after 12 months.	[61]
**Zinc (Zn^2+^)**	To evaluate the remineralization potential of various mouthwashes along with determining their effectiveness in controlling plaque and gingivitis cell response: No cytotoxic effects were observed in the test.	Mouthwash to control plaque and gingivitis.	**Remineralization:** The results from the plaque index and the gingival index score revealed reduced values when Zn-HA mouthwash was used. Moreover, DIAGNOdent analysis demonstrated better remineralization in the Zn-HA mouthwash treatment group in terms of its mineral content in contrast to the other groups (F and chlorhexidine).	[62]
**Strontium (Sr^2+^)**	Sr^2+^-substituted HA was prepared in two different concentrations (25% and 50%) and evaluated for its enamel remineralization potential using micro-indentation testing.	Biomaterial for enamel remineralization and enhanced hardness.	**Remineralization:** SEM: Spherical morphology and fewer open tubules were observed in the treated groups revealing mineral deposition. The Sr-HA samples demonstrated the highest mineral deposition. EDX: Ca and P were found in the untreated samples while Sr^2+^ traces were found in the Sr^2+^ HA groups. AFM: The treated groups demonstrated mineral deposition layers while the untreated groups demonstrated rough surfaces. The Sr^2+^ HA samples demonstrated more particle aggregation and therefore resulted in the formation of clusters rather than separate individual particles. 25% Sr^2+^ HA proved to be a better material for enamel remineralization. Microhardness: The samples treated with 50% Sr^2+^ HA revealed significantly higher hardness values in contrast to the other groups. **Biological performance:** Cell response: No cytotoxic effects were observed in the test samples.	[43]
**Zinc (Zn^2+^)**	Zn-HA toothpaste was compared with F toothpaste in terms of their potential to repair eroded enamel.	Anti-erosive toothpaste	**Remineralization:** The Vickers micro-hardness test was used to evaluate the enamel hardness. The surface hardness of the eroded enamel samples significantly increased when exposed to Zn-HA toothpaste in contrast to F toothpaste.	[63]
**Zinc (Zn^2+^)**	To evaluate the effect of chitosan-based risedronate/Zn-HA intrapocket dental film for the treatment of periodontitis in a rat model.	Biomaterial for treating periodontitis.	**Evaluation of bone formation:** On the basis of alkaline phosphatase activity, osteocalcin expression, and bone mineral density, it was concluded that the treatment with the newly formed scaffold effectively reduced the destruction of alveolar bone, thereby significantly contributing to periodontal healing.	[64]
**Zinc (Zn^2+^)**	To evaluate the remineralization and antibacterial activity of Zn-HA when incorporated into alendronate-grafted polyacrylic acid (ALN-PAA).	Biomaterial for remineralization and antibacterial agents.	**Remineralization:** Surface microhardness was significantly improved. Newly regenerated nanorods at the surface were observed via SEM analysis. **Biological performance:** Antibacterial response: Higher antibacterial activity against S.mutans was observed in the ZnHA-ALNPAA group in contrast to using HA alone.	[65]
**Fluoride (F^−^)**	To evaluate the bond strength of the cement after incorporation of nano-fluorohydroxyapatite (n-FHA) or nano-fluorapatite (n-FA).	As a cement to enhance the bond strength.	**Mechanical Properties:** Micro-tensile bond strength (µTBS) and shear bond strength (SBS). The nFA group exhibited higher SBS and µTBS values in comparison to nFHA.	[66]
**Fluoride (F^−^)**	To evaluate the efficacy of F-HA in the demineralized enamel adjacent to orthodontic brackets.	As a remineralising agent.	**Remineralization:** Lesion depth was analyzed using a polarized microscope. The F-HA-treated group revealed less demineralized depth in contrast to the control group (no prevention treatment).	[67]
**Zinc (Zn^2+^)**	To evaluate the remineralization potential of Zn-HA toothpaste on eroded enamel in contrast to the following groups: (Control: no treatment and F toothpaste).	Anti-erosive toothpaste	**Remineralization:** SEM analysis revealed irregular patterns and honeycomb-appearance structures after acid challenge. The group exposed to Zn-HA demonstrated deposited material on the surface, therefore revealing maximum protection after exposure.	[68]
**Silicone (Si^4+^)**	To evaluate the remineralization capacity of dental composites containing silanized Si-HA particles charged with NaF.	Dental composites for remineralization.	**Remineralization:** Micro-CT: The Si-HA particles charged with NaF when compared with other groups (SilF, Sil, F, and control: untreated) demonstrated an efficient reduction in the depth of artificially created lesions, as observed by micro-CT. Micro-hardness: No statistically significant difference was observed when the samples were evaluated for hardness.	[69]
**Silver (Ag^+^)**	To evaluate the fabrication of Ag-HA scaffolds in preventing the osteomyelitis during dental bone surgery.	Scaffold to prevent osteomyelitis.	**Biological performance:** Antibacterial response: The Ag-HA-substituted scaffolds presented antibacterial activity against Gram-positive and negative bacteria, but it appeared to be more effective against *S. aureus*. Cell response: Toxicity was linked to the type of cell line and concentration of Ag^+^ applied. Ag^+^ demonstrated minor toxicity against fibroblasts when 10 mol % Ag was utilized. However, when tested against mesenchymal stem cells, no toxicity was observed.	[70]
**Silicone (Si^4+^)**	To synthesize nano composite Si-HA-glycerohydrogel and to evaluate its remineralization potential.	Nanocomposites for remineralization.	**Remineralization:** AFM: Atomic force microscopy (AFM) confirmed the remineralization of human teeth. A reduction in surface roughness and a smoother enamel surface was revealed after immersion of the samples in Si-HA-glycerohydrogel. EDX: EDX analysis demonstrated an increase in the concentration of the Si in human enamel. Hardness: Vickers hardness testing indicated an increase in the hardness values in the test treatment group.	[40]
**Magnesium (Mg^2+^)**	To evaluate the fabrication of a porous scaffold with an aim to regenerate the periodontal tissue using nanofibers encapsulated with Mg-HA nanoparticles in 3D polyvinyl alcohol and bromelain. The aim was to enhance the mechanical properties and ultimately support the periodontal ligament.	Scaffold for periodontal tissue regeneration.	**Mechanical Properties:** The nanofiber-coated scaffold demonstrated excellent tensile strength as the unidirectional alignment of the fibers had enhanced interaction with the scaffold. Increased cell attachment/adhesion was observed. **Biological Performance:** Cell response: Biocompatibility was performed on human fibroblasts using live/dead assay and a greater number of live cells were observed on the scaffold. Antibacterial response: A bactericidal effect was observed when Gram-positive bacteria were exposed while no difference was observed for Gram-negative bacteria. Angiogenic analysis: An angiogenic study using ovo-cam assay was carried out and a newly formed capillary network was observed. The aortic arch assay showed a prominent ring emerging from micro-vessel, therefore confirming its angiogenic property, which is an important parameter for wound healing.	[71]
**Strontium (Sr^2+^)**	To develop a dental adhesive using Sr^2+^ substituted HA to enhance the radiopacity and mechanical properties. The groups were assigned in accordance with the thermal treatment provided. HA-Sr: 0 h. HA-Sr: 2 h. HA-Sr: 5 h.	Dental adhesive to enhance radiopacity and mechanical properties.	**Mechanical properties:** Three-point bending test: a slight impairment of mechanical properties was observed for the substituted sample. Degree of conversion: The degree of conversion was evaluated using Raman spectroscopy after light curing (ratio of cured: uncured): No statistically significant difference was obtained between the groups. Crystallinity: A highly crystalline phase was observed when the samples were treated with the HA-Sr: 5 h hydrothermal treatment. Radiopacity: Higher radiopacity was obtained for the substituted samples when compared to the control group (0 h).	[72]
**Zinc (Zn^2+^)**	To determine the efficacy of ZnCO_3_^2−^ HA solution in reviving the enamel hardness and, subsequently, its effect on the adhesion around the orthodontic brackets	Dental adhesive to enhance the hardness around orthodontic brackets.	**Remineralization and Mechanical properties**: The Vickers hardness and bond strength demonstrated higher values after exposure to the Zn-HA sample.	[73]
**Zinc (Zn^2+^)**	To determine the efficacy of Zn-HA toothpaste in reducing dentinal hypersensitivity and tooth whitening.	Toothpaste to reduce dentinal hypersensitivity and enhance tooth whitening.	**Dentinal hypersensitivity:** Patients with dentin hypersensitivity reported a reduction in cold stimulus after using the toothpaste for 4 weeks. **Tooth whitening:** A smoother and whiter tooth surface was also visualized.	[74]
**Silver (Ag+)**	To develop a bioceramic dental filling material with antibacterial and antibiofilm potential.	Dental filling material with antibacterial properties.	**Biological performance:** Antibacterial response and antibiofilm activity: Most potent inhibition zones were revealed in the Ag-HA sample when tested against *S. aureus* and *Candida albicans*. Ag-HA demonstrated excellent disinfectant potential.	[75]
**Silver (Ag+)**	To develop a nanofibrous filler using HA nanowires coated with polydopamine (PDA) and, subsequently, incorporating Ag in order to form (HA-PDA-Ag) nanowires.	As a nanofibrous filler to enhance reinforcement and to provide adequate antibacterial activity.	**Mechanical Properties:** The flexural strength and flexural modulus were increased. **Biological performance:** Antibacterial response: A significantly increased antibacterial activity was observed when tested against *S. mutans*. Cell response: Cytotoxicity analysis was performed on fibroblasts. Insignificant cytotoxicity was observed, and cells proliferated.	[76]
**Zinc (Zn^2+^)**	To investigate antimicrobial activity, conduct a biocompatibility analysis of Zn-HA, and to compare with free HA for use in dental applications.	Biomaterial for antibacterial activity.	**Biological performance:** Antibacterial response: Antibacterial activity was analyzed using CFU and MIC methods, and the following bacterial strains were used: *S. aureus*, *E. faecalis*, *E. coli*, and *P. aeruginosa*. Bacterial adherence was inhibited when exposed to the Zn-HA sample. Cell response: Biocompatibility analysis was performed on human gingival fibroblasts. A decrease in cell viability after 72 h was observed when exposed to higher doses (125 µ/mL).	[77]
**Cerium (Ce^3+^)**	To characterize and synthesize Ce-substituted HA sodium alginate biocomposite for dental implants.	Biocomposite to enhance hardness and antibacterial activity.	**Biological performance:** Antibacterial response: Antibacterial properties were assessed against *S. aureus* using the disk diffusion method. Antibacterial activity was directly proportional to the concentration of Ce^3+^ in the substituted samples (1% Ce, 2% Ce, and 3% Ce). **Mechanical properties:** Vickers hardness testing evaluated the hardness of the samples. The greatest hardness was achieved when 2% Ce^3+^ was substituted, leading to a denser compact structure with a reduction in porosity and therefore demonstrating excellent physical support for dental implants.	[45]
**Silver (Ag^+^)**	To incorporate an antibacterial agent (Ag-HA) into orthodontic adhesives for the prevention of white spot lesions.	Orthodontic composite for enhanced antibacterial activity.	**Biological performance:** Antibacterial response: Antibacterial activity of different concentrations of 0, 1, 5, and 10% Ag-HA was assessed using the disk diffusion method and the biofilm inhibition test. The 5 and 10% Ag-HA composites exhibited the greatest antibacterial activity.	[78]
**Zinc (Zn^2+^)**	To compare the efficacy of Zn-HA and F toothpaste for remineralization and treating white spot lesions	Toothpaste for remineralization.	**Remineralization:** Dental hypersensitivity: The value of the Schiff Air Index decreased significantly in the Zn-HA treatment group after 3 months. Pain: Values on the visual analog scale significantly decreased in the Zn-HA treatment group after 30 days. Dental erosion: Basic erosive examination values demonstrated significantly higher values in the treated groups after 90 days (when compared to the baseline). However, no statistical difference between the groups was observed.	[79]
**Silver (Ag^+^)**	To evaluate the antibacterial and remineralization potential of orthodontic adhesive when incorporated with different concentrations of Ag-HA (1%, 5%, and 10% Ag-HA).	Orthodontic adhesive to enhance remineralization and anti-microbial activity.	**Mechanical Properties:** Shear bond strength (SBS): The 1% and 5% Ag-HA significantly increased the bond strength when analyzed on a universal testing machine. The 10% AG-HA revealed a decrease in bond strength. This may be due to the accumulation of nanoparticles, therefore leading to point defects that may interfere with the curing process.	[80]
**Magnesium (Mg^2+^)**	To develop a dental adhesive with Mg-HA and to evaluate its remineralization and antibacterial properties. The following groups were evaluated: Control: unmodified adhesive. 0.5% Mg-HA; 1% Mg-HA; 2% Mg-HA.	Dental adhesive for remineralization and antibacterial activity.	**Remineralization:** Degree of conversion: all the test groups demonstrated a higher degree of conversion in contrast to the control group. Bond strength: the highest micro-tensile strength values were obtained for the 0.5% sample while 2% demonstrated the lowest values. Significantly improved mechanical properties were revealed in the groups in contrast to control (better resistance to collagenase-mediated degradation). Hybrid layer formation: SEM/TEM analysis was performed at the interface. A thicker and uniform hybrid layer was observed in all the test samples with adhesives in contrast to the control group. The formation of resin tags was also revealed. A more pronounced dentin interface was observed in the 0.5% and 1% samples. Nano-leakage: the 0.5% sample revealed an absence of nano-leakage, hence demonstrating better dentin bond integrity. Calcium assay: The 0.5% and 1% samples demonstrated enhanced intrafibrillar nucleation (greater calcium centers within the collagen fibril). **Biological performance:** Antibacterial response: The percentage of live bacteria (*S. aureus*) significantly decreased as the concentration of the Mg^2+^ in HA samples increased.	[34]

XRD (X-ray diffraction); SEM (scanning electron microscopy); and TEM (transmission electron microscopy).

**Table 2:** Applications of co-substituted ions in dentistry.

Co-Substituted Ions	Aim of the Study	Dental Applications	Outcomes	Reference
**Zinc (Zn^2+^)** **Carbonate (CO_3_^2−^)**	To evaluate the effectiveness of various anti-erosive toothpastes.	Toothpaste as an erosive agent.	**Remineralization:** ZnCO_3_^2−^-HA was not as effective in reducing erosive tissue loss when compared with the NaF- or Sn-containing toothpaste.	[81]
**Zinc (Zn^2+^)** **Carbonate (CO_3_^2−^)**	To evaluate the deposition of ZnCO_3_^2−^HA on dental composite restoration in patients and to compare it with fluoride.	Toothpaste as a remineralizing agent.	**Remineralization:** SEM/EDS analysis was performed to determine the levels of Ca, P, and silicone in the two groups (ZnCO_3_^2−^HA and F). Toothpaste containing ZnCO_3_^2−^HA revealed higher Ca deposits in contrast to the F group.	[82]
**Zinc (Zn^2+^)** **Carbonate (CO_3_^2−^)**	To evaluate the efficacy of toothpaste for reducing dentin hypersensitivity.	Toothpaste for reducing dentin hypersensitivity.	**Dentinal hypersensitivity:** The airblast method on the basis of the Schiff sensitivity scale was used to evaluate dentin hypersensitivity in patients. The results revealed rapid relief after treatment following the application of the test material.	[83]
**Zinc (Zn^2+^)** **Carbonate (CO_3_^2−^)** **Magnesium (Mg^2+^)** **Strontium (Sr^2+^)** **Fluoride (F^−1^)**	To evaluate the efficacy of two substituted toothpastes for reducing biofilm: Group A: (Zn-CO-HA); Group B: FHA and Mg-Sr-HA with chitosan; Group C: Control (distilled water).	Toothpaste for reducing biofilm formation.	**Biological performance:** Antibacterial response: Both groups demonstrated significant antimicrobial activity when tested against *S. mutans*.	[84]
**Zinc (Zn^2+^)** **Carbonate (CO_3_^2−^)**	To evaluate the remineralization effect of ZnCO_3_^2−^ dentifrice and to compare it with the control group (no treatment).	Dentifrice for remineralization.	**Remineralization** EDX analysis revealed minor Ca and P deposits on the treated dentin samples. Raman spectroscopy revealed no significant difference when compared with the control group.	[85]
**Zinc (Zn^2+^)** **Carbonate (CO_3_^2−^)**	To evaluate the remineralization potential of different toothpastes.	Toothpaste for remineralization.	**Remineralization:** The microradiography results obtained in evaluating the loss in mineral content demonstrated less mineral loss when ZnCO_3_^2−^ toothpaste was used in contrast to another group (amine fluoride).	[86]
**Zinc (Zn^2+^)** **Carbonate (CO_3_^2−^)**	To prevent and contrast the erosion in dentin after using different toothpastes.	Toothpaste as an anti-erosive agent.	**Remineralization:** Dentine specimens were brushed with the toothpaste and evaluated for “relative dentin abrasion.” A contact profilometer was used to evaluate the loss of dentin (reduction in dentin loss was observed). ZnCO_3_^2−^HA treatment was the least effective when compared to the other groups (NaF-nHA, chitosan, NaF/KNO3, and AmF).	[87]
**Zinc (Zn^2+^)** **Carbonate (CO_3_^2−^)**	To compare the efficacy of different mouthwashes with the aim of preventing enamel erosion.	Mouthwash to prevent enamel erosion.	**Remineralization:** Ion releasing: The release of Ca^2+^ and PO_4_^2−^ from the remineralizing solution was evaluated using SEM and TEM images. Reduced ion release was observed. The fluoride solution demonstrated better results in contrast to the ZnCO_3_-HA-containing solution.	[88]
**Strontium (Sr^2+^)** **Fluoride (F^−^)**	To synthesize and evaluate the remineralization potential, bioactivity, and biocompatibility of the following groups of nanoparticles: HA; 5%SrHA; 10%Sr-HA; F-HA; F-5SrHAF-10SrHA.	Biomaterial for remineralization.	**Biological performance:** Cell response: The 10% F-Sr-HA demonstrated marginal toxicity in contrast to the other treatment or control groups, while other groups demonstrated non-toxic effects and enhanced cell proliferation. **Remineralization:** The ALP activity of MG63 cells was accessed as it has been linked with hard tissue calcification. A positive effect on cell growth was evaluated when Sr-HA samples were used. A further enhanced effect was observed when F was also incorporated. Therefore, the conclusion was that there was a better remineralizing ability in the F-5% Sr-HA sample in contrast to the other groups.	[44]
**Carbonate** **(CO_3_^2−^), Magnesium (Mg^2+^),** **Strontium (Sr^2+^) and** **Iron (Fe^2+^)**	To analyze the remineralization capacity of toothpaste and its ability to occlude the dentinal tubules.	Toothpaste for dentinal remineralization.	**Remineralization:** SEM: Excellent remineralization was demonstrated after acid etching, as revealed by SEM analysis, showing the formation of new 100 nm-long crystals orientated in the same direction as that of the HA in enamel. Complete occlusion of dentinal tubules was observed following SEM analysis due to the deposition of a new crystalline phase.	[89]
**Magnesium** **(Mg^2+^),** **Zinc (Zn^2+^) and ** **Fluoride (F^−^)**	To investigate the effect of adding Mg^2+^, Zn^2+^, and F^−^ on the HA crystal lattice for enamel remineralization in terms of its morphology and crystalline phase.	Biomaterial to enhance enamel remineralization.	**Enamel remineralization:** A synergistic effect on the crystallization of HA within 16 h was observed when substituted with Mg^2+^, Zn^2+^, and F^−^, therefore suggesting its possible use for enamel remineralization.	[33]
**Zinc (Zn^2+^)** **Strontium (Sr^2+^)**	To enhance the remineralization potential and antibacterial activity of photocurable dental composites with an intent to decrease the probability of repair failure (which arises due to the polymerization shrinkage in photocurable composites) and may arise as a result of secondary caries.	Dental composite to enhance remineralization and antibacterial activity.	**Biological Performance:** Antibacterial response: The Zn-HA samples demonstrated superior antibacterial properties against *S. aureus* in contrast to Sr-substituted samples. Cell response: Biocompatibility analysis indicated the non-cytotoxic effects of Zn-HA samples similar to the control group (fillers with HA). These data supported their safe use in dental composites. **Remineralization:** Sr-HA revealed superior remineralization potential in contrast to other groups when analyzed under SEM, demonstrating the highest mineral deposition.	[90]
**Silver (Ag^1+^), Carbonate** **(CO_3_^2−^)**	The development of a biocompatible carbonate hydroxyapatite (CHA)-based dental implant with antibacterial (which prevents implantitis) and bioactivity properties. Different concentrations of Ag^+^ were used (0.005, 0.01, and 0.015 mol).	Biomaterial to prevent implantitis.	**Biological performance:** Antibacterial response: The antibacterial activity of the Ag^+^ demonstrated a strong effect against Gram-positive bacteria such as S.aureus. Cell response: Osteoblast cell lines were used to analyze the biocompatibility of Ag^+^. The cytotoxicity decreased even at the highest Ag^+^ concentration. CHA-Ag-15 had the highest antibacterial activity and lowest cytotoxicity.	[46]

EDS (energy dispersive spectrometer); SEM (scanning electron microscopy); and TEM (transmission electron microscopy).

## 4. Applications of HA-Ion Substitutions in Dentistry

**Figure 4:**
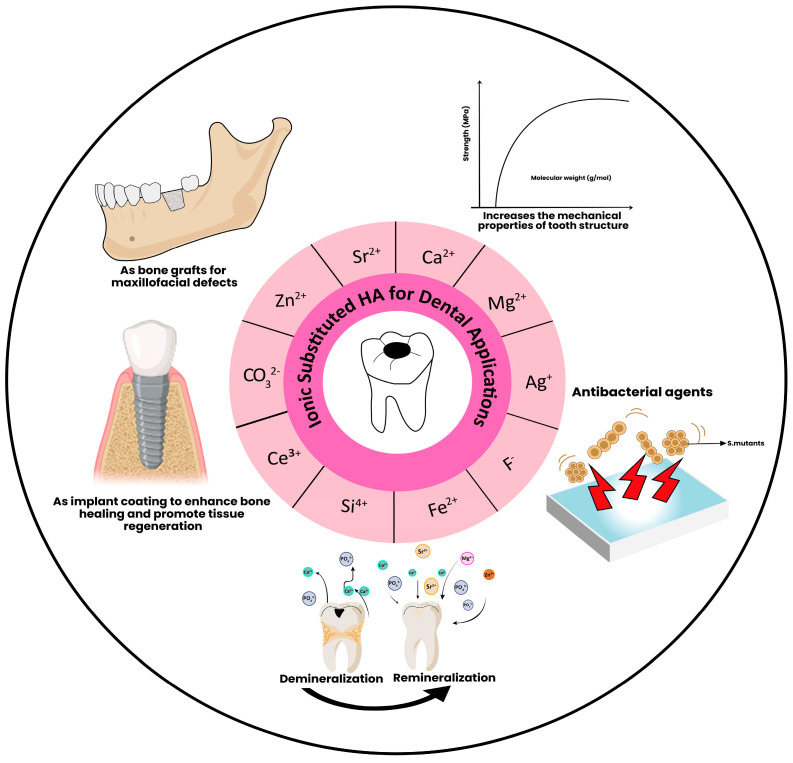
Dental applications of ionic-substituted HA.

Substituting ions into the HA structure offers potential for numerous applications in dental practice. Indeed, as is highlighted here, various in vitro investigations have been performed using substituted materials, with the aim of exploring their potential use to augment antibacterial activity, enhance remineralization, and treat dentinal hypersensitivity or enamel erosion. Figure 4 summarizes the dental applications of substituted hydroxyapatite.

### 4.1. Enamel Remineralization and Repair

The rods in enamel comprise HA and are arranged in a tightly packed crystal structure that enables the tissue to withstand significant masticatory forces by providing rigidity and overall strength to the tooth. Caries is a prevalent, non-communicable disease that results when there is an equilibrium imbalance between an acidogenic biofilm and tooth mineral, which consequently results in a demineralization of the dental hard tissue. With the advancement in technology and our understanding of disease progression, traditional surgical treatment is being replaced with preventative and remineralization approaches of the tooth structure that utilize the topical application of fluoride [91] or nano HA-based treatments [18,92]. The release of fluoride from the lattice structure can significantly reduce caries susceptibility and progression, subsequently stabilizing the crystal lattice as a result of fluorapatite formation. Notably, as fluoride-based treatment approaches at a population level have reached a plateau, HA-based treatments have gained widespread attention in filling the gap in a remineralization efficacy that utilizes fluorides [93]. In vitro investigations along with clinical studies have demonstrated the remineralization potential of HA nanoparticles; however, this material alone lacks antimicrobial and appropriate mechanical properties. Consequently, researchers have attempted to generate HAs with these tripartite effects [94].

An in vitro study undertaken by Krishnan investigated the effects of incorporating Sr^2+^ in a HA lattice structure with an aim to regenerate enamel after orthodontic treatment. This clinical treatment is invasive and can lead to the erosion of the tooth surface, which promotes colonization of acid-producing bacteria and subsequently leads to the formation of incipient caries lesions. Secondly, the orthodontic bonding procedure requires the use of demineralizing agents, which can also result in cavitation, therefore making the restorative treatment extremely challenging. Various remineralization agents, such as fluorides, calcium supplements, or synthetic HAs have documented limitations such as the inadequate particle size, strength of HA or the poor biocompatibility of fluorides. Consequently, this has necessitated the development of a resilient material to overcome these problems, which has been successfully achieved when Sr^2+^ is substituted into the HA lattice structure. The comparative analysis demonstrated the superior remineralization potential of Sr-HA when compared with conventional treatments [43].

To produce a composite with the potential to release Ca^2+^ and PO_4_^3−^, Reis and colleagues incorporated Si-HA into composites for enamel remineralization and used the process of silanization to improve the chemical interaction of the organic and inorganic matrix. The Si-HA material functioned as a reservoir for NaF, subsequently allowing F release. However, the results revealed a reduction in the remineralization potential as a consequence of silanization as it entrapped the F particles, which were primarily responsible for remineralization [69]. However, the results revealed a reduction in the remineralization potential as a consequence of silanization as it entrapped the F particles that were primarily responsible for remineralization [69].

In another study, Daood et al. explored the incorporation of Mg-HA into dental adhesives to enhance their remineralization and antibacterial properties [34]. SEM and TEM data revealed the formation of a thick hybrid layer along with resin tags, while bond strength studies revealed significantly improved bond strength [34]. Periodontitis, a major global healthcare burden affecting 20% of the adult population and often leading to tooth loss, underscores the need for innovative treatments. Addressing this issue, Shoba et al. developed a nanofibrous scaffold combining Mg-HA particles with a plant proteolytic enzyme bromelain. This scaffold aims to improve mechanical properties, provide adequate support to the periodontal tissue, and enhance angiogenesis to facilitate tissue repair [71].

### 4.2. The Antimicrobial and Antibiofilm Activity of Substituted HA

The treatment of infections around the bone implant after reconstruction surgery remains challenging [95,96]. Currently, this necessitates the need for antibiotic therapy to support the body’s natural defense mechanisms, which is usually delivered via the oral route. The limitations of oral antibiotics include decreased drug delivery at the infection site, requiring the need to increase the dose for effectiveness, which may lead to toxic effects. Therefore, there is a need to develop materials with enhanced antibacterial properties, which may eliminate the need for additional antibiotic treatment. Numerous in vitro investigations have been performed that have incorporated ions such as Ag^+^, Zn^2+^, or Cu^2+^ into HA and have demonstrated notable improved properties, including inhibiting the growth of a wide range of bacterial species. Interestingly, only a relatively small amount of information is available in the literature regarding the hypothetical antibacterial mechanism of these ions. One proposed mechanism is the disruption of bacterial DNA replication, and this occurs when the ion penetrates the cell and inhibits ATP production. Another proposed mechanism is due to the activation of reactive oxygen species when the cells come into contact with the ions. The oxygen radical generated reacts with the constituents of the bacterial cell wall and membrane, resulting in irreversible bacterial damage. The third proposed mechanism was linked to the inhibition of the transportation of protons as a result of the accumulation of ions in the bacterial cell wall. This subsequently modified the cell wall permeability, which ultimately resulted in cell death [97,98].

A study using substituted Ag^+^ with carbonate HA aimed at treating peri-implantitis demonstrated the successful inhibition of bacterial growth. However, biocompatibility results were of concern as they demonstrated a decrease in the viable cell count as the concentration of undissolved particles increased. However, this effect was not directly related to the presence of Ag^+^ [46], justifying the safe usage of the material.

### 4.3. Dental Bone Regeneration

In the recent decade, the use of titanium-based dental implants has significantly increased in dental practice [99]. However, despite the relatively high success rate, on some occasions, there may be insufficient bone formation after implant placement, which can lead to implant failure [99], especially in osteoporotic patients [100]. Consequently, the endosseous metal component of the dental implant can require a coating to enhance its osteoconductivity [101]. HA is a frequently used bioactive material for coating layers on prosthetic devices to enhance the process of bone healing [101]. According to ISO standards, for surgical implants, a coating is required to have a crystalline and an amorphous phase to prevent rapid dissolution and to promote osteointegration, respectively. Secondly, its elemental composition should be similar to the mineral phase of bone while maintaining a strong adhesion to prevent mechanical failure [102]. Currently, calcium phosphates fabricated using plasma spraying are commercially available. However, recently, the substitution of HA with various ions for implant coating has attracted the attention of dental researchers. These novel coatings hold significant promise for the future by potentially enhancing implantation processes, thereby accelerating bone healing and promoting tissue regeneration [20]. Ion substitution provides increased resistance to corrosion and augments the strength, therefore making it more appropriate to be used in load bearing areas. A study by Vladescu et al. demonstrated improved hardness and elastic modulus when HA was substituted with various ions [103]. Additionally, Balakrishnan compared the durability and confirmed the enhanced durability of substituted HA in contrast to pure HA [104]. Meanwhile, Manzoor et al. revealed a direct relation between the concentration of the ion substituted with the young modulus of the newly formed material [105]. Therefore, as discussed, the applicability of HA may be enhanced when substituted with various ions. Table 1 and Table 2 summarize the recent studies of substituted HA in dental bone regeneration.

## 5. Biocompatibility

HA is an essential material in the human body that has the potential to be modified in order to improve various grafting approaches regarding their physio-chemical or biological parameters. However, these modifications to the chemistry of HA can raise biocompatibility concerns. Table 1 highlights the key findings that relate to the toxicity of various ion-containing HAs.

Krishnan and colleagues performed a toxicity analysis using L929 fibroblast cell lines with two different concentrations of Sr^2+^ (25 mol% and 50 mol%). Data demonstrated an improved biological response (i.e., an increase in viable cells) when Sr^2+^ was incorporated in HA in contrast to unmodified HA [43]. Consistent findings were also reported in an investigation where cell viability was assessed using Sr^2+^ at two different concentrations (5% Sr-HA and 10% Sr-HA) and F^−^ (F-5% Sr-HA and F-10% Sr-FHA) using an MTT assay of osteoblast cell lines. Positive effects on cell proliferation were identified for all the tested groups; however, a slightly decreased cell viability was obtained for the F-10% Sr-FHA group. Consequently, it was concluded that Sr^2+^ did not exert toxic effects and had the potential to increase cell responses and viability [44].

Paterson et al. explored the toxicity effects of Ag^+^ on fibroblasts and mesenchymal stem cells (MSCs) at three different concentrations (2%, 5%, and 10 mol% Ag-HA). Some indication of toxicity in fibroblasts was identified for the 10% mol Ag-HA exposure group while MSCs did not demonstrate any cytotoxic effects at this concentration [70]. Similar findings were also reported in another investigation, where non-cytotoxic effects on pre-osteoblast cells were identified when Ag^+^ was used at higher concentrations [46].

In vitro investigations evaluating Zn^2+^ toxicity revealed a non-cytotoxic effect of Zn^2+^ when used in combination with Sr^2+^ once incorporated in composites at different concentrations (2%, 6%, and 8%). These data indicated the safe use of these ions in dental treatments [90]. In another study, no significant difference in cell viability after 24 h incubation was demonstrated, although a marginal decrease was observed following 72 h of exposure with a dose of Zn^2+^ at 31.25 μg/mL. Notably, the cell membrane remained intact, although relatively high doses (125 μmL) did affect cell adhesion. Therefore, it was concluded that the benefits of zinc substitution are concentration-dependent. Indeed, at high concentrations, Zn^2+^ may induce an anti-inflammatory response while a reverse effect is observed if used at low concentrations [77].

## 6. Conclusions

HA is a widely used material for tooth remineralization and bone tissue engineering due to its resemblance with natural osseous tissue, and it is strongly recommended for its osteogenic and osteo-conduction properties, while its high biocompatibility makes it an ideal candidate for bone tissue engineering. However, mechanical strength and bioactivity are a few of the limitations that makes the introduction of ions into the HA lattice structure a reasonably favorable approach that can be used to modify HA properties. This review provides evidence that ion substitution in a HA lattice structure may enhance physicochemical mineralization, consequently resulting in enamel remineralization or repair when applied to the tooth structure. Moreover, the substituted ions have the potential to enhance the material’s antimicrobial properties and to facilitate bone regeneration.

There is an immense need for the optimization of methods used for ion substitution and for finding the effect of each type of ion in the human body, especially on the basis of its concentration. Thus, it is highly recommended for future research in order to develop an ideal biomaterial. This novel approach can ultimately be used for commercial translation into various dental products such as toothpaste or mouthwashes with subsequent clinical evaluation of those novel oral care products. Recently, HA obtained from natural sources such as bovine or fish bones, eggshells, or seashells has become an increasing area of research. Therefore, it would be of interest to investigate the interrelation between the HA obtained from these natural resources and to introduce substituted ions into the lattice structure of HA. This innovative approach would also address various concerns such as sustainability and responsible resource management, while the cost-effective approach would make the products accessible to a broader population.
